# An iTRAQ-Based Proteomics Approach to Clarify the Molecular Physiology of Somatic Embryo Development in Prince Rupprecht's Larch (*Larix principis-rupprechtii* Mayr)

**DOI:** 10.1371/journal.pone.0119987

**Published:** 2015-03-17

**Authors:** Jian Zhao, Hui Li, Shuangbin Fu, Bo Chen, Wenting Sun, Junqi Zhang, Jinfeng Zhang

**Affiliations:** National Engineering Laboratory for Tree Breeding, Key Laboratory of Genetics and Breeding in Forest Trees and Ornamental Plants, Ministry of Education, The Tree and Ornamental Plant Breeding and Biotechnology Laboratory of State Forestry Administration, College of Biological Science and Biotechnology, Beijing Forestry University, Beijing 100083, China; Institute of Botany, Chinese Academy of Sciences, CHINA

## Abstract

Prince Rupprecht's larch (*Larix principis-rupprechtii* Mayr) is a native high-value forest tree species in North China whose clonal propagation through somatic embryogenesis (SE) has the potential to rapidly capture the benefits of breeding or genetic engineering programs and to improve raw material uniformity and quality. To date, research has focused on clarifying the molecular mechanism of SE, but proteomic studies are still in the early stages. In this study, isobaric tags for relative and absolute quantitation (iTRAQ) analysis was performed on three developmental stages of SE in *L*. *principis-rupprechtii* in an attempt to identify a wide range of proteins that are regulated differentially during this process. Proteins were extracted and analyzed from the pro-embryogenic mass (PEM), globular embryo (GE), and cotyledon embryo (CE) stages of embryo development. We detected 503 proteins in total and identified 96 proteins expressed differentially during different developmental stages. The identified proteins were analyzed further to provide information about their expression patterns and functions during SE. Four clusters of proteins based on shared expression profiles were generated. Functional analysis showed that proteins involved in primary metabolism, phosphorylation, and oxidation reduction were upregulated during somatic embryo development. This work provides novel insights into the process of larch embryo development in vitro and a basis for further study of the biological process and opportunities for practical application of this knowledge.

## Introduction

Prince Rupprecht's larch (*Larix principis-rupprechtii* Mayr) is an important native coniferous tree species in North China that plays a critical role in reforestation programs and commercial use due to its wide ecological plasticity, rapid growth, and desirable wood product. However, its utilization is hindered to some extent by the restrictions of conventional breeding and propagation methods.

Somatic embryogenesis (SE) provides an excellent illustration of plant totipotency by virtue of its ability to produce morphologically and developmentally normal embryos and even whole plants from somatic cells in culture through a process resembling zygotic embryogenesis [1]. Clonal propagation of high-value forest trees such as *L*. *principis-rupprechtii* through SE has the potential to rapidly capture the benefits of breeding or genetic engineering programs and to improve raw material uniformity and quality [2]. Therefore, understanding the molecular mechanisms underlying SE can provide insight into developmental and metabolic regulation, and the signaling systems that integrate these processes.

Previous research has focused on the characterization of genome-wide expression of genes at the mRNA level. Numerous genes have been identified that are specifically expressed during SE including the *SERK* [3], *LEAFY COTYLEDON* [4,5], *BABY BOOM* [6], and *WUSCHEL* [7] genes. The rapid development of “omics” methodologies in recent years has provided unprecedented opportunities to describe the embryogenesis process in terms of morphological and cytological events and to better understand the regulation of this developmental pathway at the molecular level. However, even though proteins are directly responsible for the functions and phenotypes of cells, protein abundance does not always correlate well with mRNA abundance. Therefore, the use of proteomics methods has provided a powerful approach to explain more directly the process of SE. In fact, many studies on SE have been conducted in the proteomes of economically important plant species such as maize (*Zea mays* L.) [8,9], *Medicago truncatula* [10,11], grapevine (*Vitis vinifera*) [12,13], *Cyclamen persicum* [14,15], orange tree (*Citrus sinensis*) [16], date palm (*Phoenix dactylifera* L.) [17], and tamarillo (*Cyphomandra betacea*) [18].

To date, increasing studies have focused on protein expression during embryogenesis in conifers. Lippert et al. [19] indicated some important proteins that involved in SE in white spruce. In addition, a differential proteome analysis of ECs with different maturation capabilities in Brazilian pine [20] was carried out. More recently, Teyssier et al. [21] have made progresses on global DNA methylation and proteomic changes during somatic embryo maturation in hybrid larch. Even so, proteomic study on SE in conifers is still in the early stage. Moreover, it is also an unexplored area to focus on the process of SE in *L*. *principis-rupprechtii* at the protein level.

The aim of this study was to identify a wide range of proteins that are regulated differentially during somatic embryo maturation in *L*. *principis-rupprechtii*. In this study, isobaric tags for relative and absolute quantitation (iTRAQ) combined with liquid chromatography–tandem mass spectrometry (LC-MS/MS) were employed to assess protein expression profiles quantitatively in somatic embryos during the pro-embryogenic mass (PEM), globular embryo (GE), and cotyledon embryo (CE) developmental stages. This analysis identified four temporal expression patterns (clusters) of differentially expressed proteins. Proteins of the four clusters belonged to different functional categories and clearly indicated dynamic changes in the proteins involved in the development of *L*. *principis-rupprechtii* somatic embryos. This work provides novel insights into the process of larch embryo development in vitro and a framework both for study of the biological processes as well as opportunities for practical application of this knowledge.

## Materials and Methods

### Tissue culture and sample collection

The BL2e cell line established at the National Engineering Laboratory for Tree Breeding (BJFU, Beijing, China) was selected for this proteomic study. The embryogenic material was subcultured biweekly on maintenance medium. Samples collected from separate clumps of proembryogenic masses (PEM) were considered to be single biological replicates. Proteomic sample collection was according to Lippert’s method [19]. Briefly, 1 g of fresh PEM (five replicates) was suspended by vigorous shaking for 3 min in 15 mL of liquid maintenance medium without plant growth regulator (PGR), and a 5-mL aliquot of the suspension was then spread evenly over a filter paper disc (No. 2, 70 mm diameter; Whatman, Maidstone, Kent, UK) using vacuum. The filter paper with distributed PEMs was then placed on solid maintenance medium for 7 days. At this point, tissue was designated as PEM stage, and was either collected or transferred to transition medium with 1% activated carbon (AC) without PGR for somatic embryo maturation and further sample collection. After 7 days on the transition medium, the tissue-carrying filter paper was transferred onto maturation medium [basal medium supplemented with 60 μM abscisic acid (ABA) and 6% sucrose and solidified with 0.5% Phytagel]. All materials were cultivated in darkness at 25 ± 1°C. After ∼1 week, globular-shaped embryos designated as GE stage were harvested. After 6 weeks of culture, CEs with no less than five cotyledons were collected and designated as CE stage. Harvested samples were frozen immediately in liquid nitrogen and stored at—80°C until use.

### Protein extraction, in-gel digestion, and iTRAQ labeling

Frozen samples of three stages were ground in liquid nitrogen to a fine powder and suspended in lysis buffer containing 8 M urea, 30 mM HEPES, 1 mM phenylmethyl sulfonyl fluoride (PMSF), 2 mM EDTA, and 10 mM dithiothreitol (DTT). Samples were then sonicated for 5 min and centrifuged at 20,000 × g for 30 min. Protein concentration was determined using the Bradford assay [22]. 1D SDS-PAGE was performed to separate proteins (50 μg of each sample) on the first dimension. Sixteen fractions in each lane were divided and carefully excised from the gel lanes and transferred to 2-mL centrifuge tubes. Gel fractions were destained with 50% acetonitrile (ACN) and 25 mM triethylammonium bicarbonate (TEAB) overnight and subsequently dehydrated with ACN until bleaching. Proteins were reduced with 10 mM DTT at 56°C for 1 h, alkylated with 55 mM iodoacetamide (IAM) at room temperature for 1 h, and then centrifuged at 20,000 × g for 30 min. The resulting pellets were washed twice with 25 mM TEAB and twice with destaining solution, and ACN was added to repeat the dehydration step. For protein digestion, enzyme stock solution (1 μg/μL trypsin) in 150-fold dilution was added to the pellets followed by overnight incubation with 25 mM TEAB at 37°C. Extraction was then performed with destaining solution for 30 min and ACN until completely dewatering. The extracted liquid from the previous three extraction steps was collected together, and peptides were lyophilized and stored at—80°C. Lyophilized peptides from each sample were subsequently dissolved in 20 μL of 50% TEAB and mixed with isopropanol by vortexing for 1 min. iTRAQ experiment was performed in double duplex manner using the iTRAQ Reagent-8 Plex Multiplex Kit (Applied Biosystems, Foster City, CA, USA) according to the manufacturer's instructions, where “PEM” was labeled with 115 and 116, “GE” was labeled with 117 and 118, and “CE” was labeled with 119 and 121, and incubated at room temperature for 2 h. Following labeling and quenching, the six iTRAQ-labeled peptides were pooled together and lyophilized.

### RP nano LC-MS/MS

Prior to MS identification, peptide fractions were dissolved in 0.1% FA with 5% ACN and further separated on a Dionex UltiMate 3000 high-performance LC system with a built-in microfraction collection option in its autosampler and UV detection (Thermo-Dionex, Sunnyvale, CA, USA). The system was equipped with an isocratic pump working at 20 μL/min (0.1% FA and 5% acetonitrile in water) for quick sample loading into an enrichment column (Acclaim PePmap 100, 75 μm × 2 cm, nanoviper, C18, 3 μm, 100A; Thermo Scientific, Waltham, MA, USA). Subsequently, peptides previously trapped on the enrichment column were eluted and further separated on an analytical column (C18, 100 mm × 75 μm, 5-μm particles) using a gradient with increasing concentrations of solvent B (0.1% FA in ACN; solvent A contained 0.1% FA in water) at a flow rate of 400 nL/min (0 min, 5% B; 10 min, 5% B; 40 min, 30% B; 45 min, 60% B; 48 min, 80% B; 55 min, 80% B; 58 min, 5% B; 65 min, 5%B), combined with a Q-Exactive mass spectrometer (Thermo Scientific), which is a hybrid quadrapole and Q-trap mass spectrometer. Data were acquired using a data-dependent acquisition mode in which, for each cycle, the 15 most abundant multiply charged peptides (2+ to 4+) with an *m/z* between 400 and 1800 were selected for MS/MS with the 15-s dynamic exclusion setting.

### Protein identification and data analysis

For peptide data analysis, raw mass data were processed using Proteome Discover 1.3 software (Thermo Scientific, Waltham, MA, USA) and searched using in-house MASCOT software 2.3.0 (Matrix Science, London, UK) against the green plant (28,783 entries, Jan. 2014) and Pinaceae (39,796 entries, Jan. 2014) protein databases downloaded from the Swissprot and Arabidopsis (TAIR10, 35386 entries, Jan. 2014) protein databases. Searches were performed as our earlier iTRAQ study [23] using the following criteria: full tryptic specificity was required with tolerance set at one missed cleavage; carbamidomethylation of Cys was set as fixed modifications; iTRAQ 8-plexs modification of K, Y, N-terminus, Gln–pyro–Glu of the N terminus, deamination of the N terminus, and oxidation of Met were set as variable modifications; and peptide and fragment mass tolerance values were 15 ppm and 0.02 Da, respectively. All reported data were based on 95% confidence intervals for protein identification as determined by a false discovery rate (FDR) less than or equal to 1%. Quantitative protein ratios were weighted and normalized using the median ratio with automatic outlier removal in MASCOT. The list of proteins obtained from the iTRAQ data was exported to Excel (S1 Dataset) and contained protein-specific information such as accession numbers assigned to each identified protein, protein scores, percent coverage, and number of peptides matching individual proteins. Changes in expression were calculated in comparison with corresponding controls based on the cumulative intensity of reported ions in each identified peptide; proteins were identified as expressed differentially with statistical significance at any time point compared to the control (PEM stage) based on two criteria: a fold-change cutoff of 1.2 relative to the control (i.e., an expression ratio greater than 1.20 or less than 0.83) and an expression ratio with a *p*-value less than 0.05. Gene Ontology (GO) analysis of the differentially expressed proteins was performed by searching UniProtKB (http://www.uniprot.org/) for protein annotation. The proteins were then classified based on their biological functions using Web Gene Ontology Annotation Plot software (WEGO; http://wego.genomics.org.cn/cgi-bin/wego/index.pl). Cluster analysis was performed to demonstrate the distribution of expression patterns among the three developmental stages [24].

### Water content assay

Fresh samples of three developmental stages (PEM, GE and CE) were weighed after harvest to determine their fresh weigh (FW). Dry weigh (DW) was determined after over-drying at 70°C for 8 h. The water content was calculated as (FW—DW)/FW and expressed as a percentage. Five biological replicates were used for each developmental stage.

### Assay of antioxidant enzyme activity

For enzyme activity assay, samples of three stages (PEM, GE and CE) were separately homogenized in 0.1M phosphate buffer (pH 7.4) forming 10% (w/v) homogenates. The homogenates were centrifuged at 4°C, 3500rpm for 10min and the supernatants were collected for determination of the activities of two antioxidant enzymes, superoxide dismutase (SOD) and catalase (CAT), using commercial kits (Nanjing Jiancheng Bioengineering Institute, Nanjing, China). Protein concentration was determined using the Bradford assay [22].SOD activity was assayed by the xanthine/xanthine oxidase method based on the production of O^2-^ anions, and the activity of SOD was expressed as units per milligrams of protein (U/mg prot). CAT activity was determined by the rate at which it caused the decomposition of H_2_O_2_ at 240nm, and the activity of CAT was expressed as U/g prot.

## Results

### Development of somatic embryos in L. principis-rupprechtii

**Fig 1:**
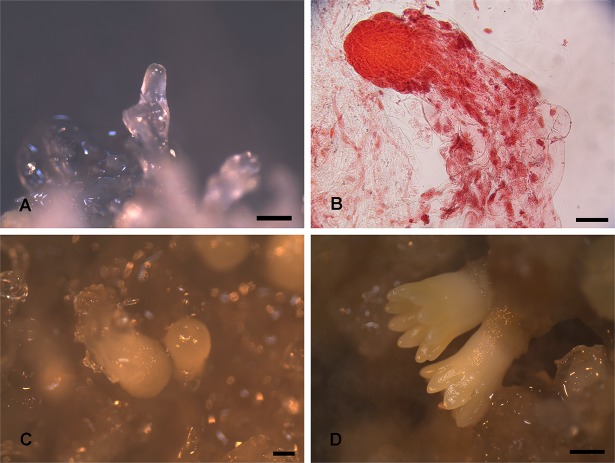
The development of cultured somatic embryos in *L*. *principis-rupprechtii*. Samples were imaged at three developmental stages: PEM (A, bar = 500μm; B, bar = 100μm), GE (C, bar = 200μm) and CE (D, bar = 500μm).

Two major phases in somatic embryo development in conifers were characterized. The first phase was the acquisition of PEMs. These PEMs were visually translucent, glossy, and mucilaginous (Fig. 1A) and were considered to be the very early stage of the immature somatic embryo as described previously by Lippert [19]. Microexamination revealed the structures of a well-defined embryonal masses consisting of clusters of cells with large nuclei and dense cytoplasm as well as a suspensor with elongated, vacuolated cells (Fig. 1B).

When transferred to ABA-supplemented medium, PEMs advanced through several distinct developmental stages associated with maturation. After 7 days of maturation, small globular-shaped embryos were visible (Fig. 1C). These embryos continued to enlarge with accompanying suspensor degeneration and finally differentiated into CEs (Fig. 1D), which could be separated readily from surrounding tissues. Further, under the system used, the somatic embryos would continue to develop and germinate into somatic seedlings.

### Overall proteome at various somatic embryo developmental stages

**Fig 2:**
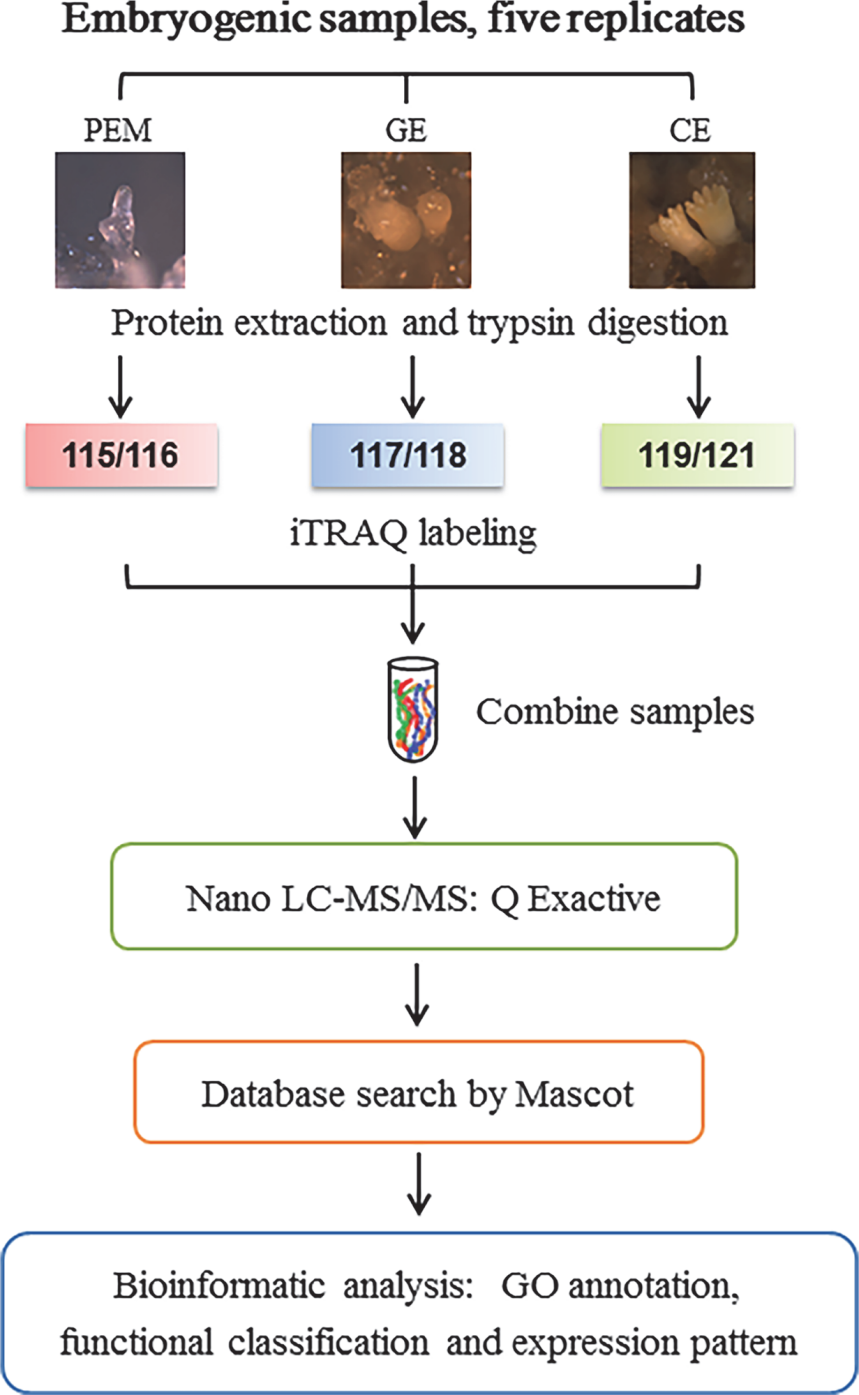
Experimental design and iTRAQ workflow used in this study. Samples were analyzed by iTRAQ coupling with nanoLC-MS/MS for examining the proteome changes during SE in *L*. *principis-rupprechtii*. Proteins were identified using MASCOT software.

To investigate the developmental changes during *L*. *principis-rupprechtii* SE from a physiological perspective, proteins of the PEM, GE, and CE stages were extracted and analyzed using an iTRAQ-based shotgun proteomics strategy. Samples were double labeled with iTRAQ tags (PEM with 115 and 116, GE with 117 and 118, and CE with 119 and 121) for higher confidence in identification. An overview of the iTRAQ experimental design and workflow is presented in Fig. 2.

A total of 801 proteins were identified based on the protein databases for other species (S1 Dataset). After discarding 298 putative uncharacterized proteins, the combined data from the three databases resulted in 503 proteins, which were assigned unambiguous descriptions. And ninety-six differentially expressed proteins were identified based on the screening criteria (Table 1).

**Table 1:** Functional classification on differential proteins with iTRAQ ratios > or < 1.2-fold.

Protein name	Accession^*a*^	PEM^*b*^	GE^*b*^	CE^*b*^	Cluster^*c*^
**Primary metabolic process**					
Triosephosphate isomerase	D5AAB7	1	0.352	7.143	2
Triosephosphate isomerase, chloroplastic	P48496	1	0.325	7.336	2
Citrate synthase	B8LLL1	1	0.969	0.579	1
6-phosphogluconate dehydrogenase, decarboxylating	E3V1H6	1	1.267	1.326	4
6-phosphogluconate dehydrogenase family protein	Q9SH69	1	0.776	1.350	4
Glyceraldehyde-3-phosphate dehydrogenase of plastid 2	Q5E924	1	0.803	0.510	1
Aldose 1-epimerase	A9NMT6	1	2.146	0.562	3
Class IV chitinase	C3VP99	1	0.409	3.559	2
Alpha-1,4-glucan-protein synthase [UDP-forming]	O04300	1	0.940	0.250	1
5-methyltetrahydropteroyltriglutamate—homocysteine methyltransferase	Q42662	1	0.963	0.402	1
Peptidyl-prolyl cis-trans isomerase	A9NLD0	1	0.781	3.226	2
Peptidyl-prolyl cis-trans isomerase	A5HIY2	1	0.556	3.077	2
**Phosphorylation**					
Nucleoside diphosphate kinase (Fragment)	P47921	1	3.493	3.269	4
Nucleoside diphosphate kinase	A9NZL7	1	2.358	3.145	4
Nucleoside diphosphate kinase 1	P39207	1	2.004	7.746	4
Nucleoside diphosphate kinase 2	Q0WUG9	1	3.205	3.247	4
Phosphatidylinositol-4-phosphate 5-kinase	F4HRM3	1	1.050	5.747	4
**Gene expression**					
60S ribosomal protein L11	P46287	1	0.185	11.664	2
40S ribosomal protein S8	P49199	1	0.499	1.853	2
40S ribosomal protein S5–2	P51427	1	0.514	2.965	2
40S ribosomal protein S13	P46298	1	0.568	1.351	2
60S ribosomal protein L13	P49627	1	0.576	0.675	1
40S ribosomal protein S3–3	Q9FJA6	1	0.728	0.830	2
60S ribosomal protein L12	O50003	1	1.299	11.443	4
40S ribosomal protein S14	Q9XEK6	1	1.309	2.479	4
40S ribosomal protein SA	Q9ZSR8	1	1.056	0.455	1
40S ribosomal protein S24	A9NKN3	1	1.575	0.597	3
40S ribosomal protein S8	A9NMR0	1	0.693	0.773	1
60S ribosomal protein L13	B8LQY1	1	0.623	0.659	1
60S ribosomal protein L6	A9NLS7	1	0.331	0.699	2
40S ribosomal protein SA	A9NV56	1	1.164	0.395	1
Ribosomal L5P family protein	P42794	1	0.170	11.628	2
Ribosomal protein L11 family protein	P50883	1	1.193	11.364	4
Ribosomal protein S11 family protein	Q9SIH0	1	1.203	2.463	4
Ribosomal protein L16p/L10e family protein	F4HUJ2	1	0.805	0.745	1
40s ribosomal protein SA	B9DG17	1	1.008	0.540	1
Elongation factor 1-alpha	O64937	1	0.667	2.916	2
Eukaryotic initiation factor 4A-3	P41380	1	1.672	0.729	3
Eukaryotic initiation factor 4A-1	P41376	1	1.047	0.560	1
Elongation factor 1-alpha (Fragment)	Q9AVT7	1	0.822	3.279	2
Elongation factor 1-alpha (Fragments)	P85915	1	0.646	0.474	1
GTP binding Elongation factor Tu family protein	F4HUA0	1	0.918	3.704	4
Eukaryotic translation initiation factor 4A1	F4JEL5	1	0.962	0.556	1
Proliferating cell nuclear antigen	O82134	1	0.738	0.442	1
Proliferating cell nuclear antigen	A9P175	1	0.797	0.431	1
**Nutrient reservoir activity**					
Legumin-like storage protein	Q40933	1	0.584	10.870	2
**Oxidation reduction**					
Catalase-3	Q42547	1	1.395	1.445	4
Catalase 3	Q2V4M4	1	1.351	1.458	4
Catalase	B8LKX4	1	1.502	1.431	4
Catalase	A9NUZ2	1	1.059	0.478	1
Superoxide dismutase [Cu-Zn]	A9NV74	1	0.902	1.499	2
Putative flavonoid 3'-hydroxylase (Fragment)	Q8RVJ5	1	5.780	0.935	3
**Cytoskeleton organization**					
Actin-51 (Fragment)	Q96483	1	1.335	0.292	1
Actin 1	C3VIW3	1	0.968	0.398	1
Actin-12	P53497	1	0.695	0.393	1
Profilin	A9NNS7	1	1.287	1.905	4
Tubulin alpha-3/alpha-5 chain	P20363	1	1.217	0.809	3
Tubulin beta-7 chain	P29515	1	1.152	0.858	3
Tubulin alpha-1	P11139	1	0.949	0.880	1
**Transport**					
ADP, ATP carrier protein (Fragment)	Q9AVT6	1	0.548	2.646	2
ARF-GAP domain 1	Q9FIT8	1	0.339	1.488	2
Endomembrane-type CA-ATPase 4	Q9XES1	1	0.901	0.647	1
Calcium-transporting ATPase, endoplasmic reticulum-type	Q42883	1	0.981	0.651	1
Importin alpha isoform 2	F4JL11	1	1.618	0.691	3
SecY protein transport family protein	Q8RWJ5	1	0.766	0.319	1
Clathrin, heavy chain	Q0WNJ6	1	0.978	0.663	1
ATP synthase subunit alpha, mitochondrial	P05494	1	2.174	1	3
ATP synthase subunit beta, mitochondrial	P17614	1	1	0.769	1
ATP synthase subunit alpha	I3VKE8	1	2.347	0.977	3
ATP synthase subunit beta	A9NUR7	1	1.224	1.499	4
ATP synthase alpha/beta family protein	D7M1G8	1	1.085	1.006	3
**Binding**					
Calmodulin	P04352	1	0.748	1.619	2
Calmodulin (Fragment)	C6FCQ5	1	0.808	1.580	2
Annexin	A9NNL2	1	1.116	0.293	1
Luminal-binding protein	Q42434	1	1.253	0.36	1
Luminal binding protein	Q40924	1	1.355	0.352	1
Glycine-rich RNA-binding protein 2	P84976	1	1.685	1.465	4
DEAD-box ATP-dependent RNA helicase 2	Q94A52	1	2.016	0.658	3
Heat shock cognate protein 70–1	F4KCE5	1	1.144	0.343	1
Regulatory particle triple-A ATPase 6A	Q9C5U3	1	2.882	0.805	3
**Nucleosome assembly**					
Histone H2B (Fragments)	Q99285	1	0.575	10.349	2
Probable histone H2B.3	Q1SU99	1	0.788	10.605	2
Histone H4 variant TH011	P62785	1	1.089	1.873	4
Histone H3	A9NMA0	1	1.255	2.463	4
Histone H2A (Fragment)	K7NMH3	1	0.359	7.194	2
Histone H4	A9NLQ1	1	1.110	2.457	4
Histone H2B	Q1H5F9	1	0.541	11.364	2
Histone H4	Q6NR90	1	1	1.862	4
Histone H3	Q0WRA9	1	1.043	1.504	4
Histone H2A.6	Q9LD28	1	0.798	1	2
**Other proteins** ^***d***^					
Ubiquitin-like protein 1	P0C033	1	1.265	5.826	4
Ubiquitin (Fragment)	A8T3H0	1	1.368	5.682	4
Polyubiquitin 10	Q3EAA5	1	1.163	5.780	4
14–3–3-like protein D	O49996	1	0.370	1.010	2
Breast basic conserved 1	F4IWP7	1	0.527	0.681	2
Embryo-abundant protein	D9IWE0	1	1.045	0.778	1

^*a*^ Protein accession numbers in Swiss-prot

^*b*^ The iTRAQ ratios for PEM, GE and CE using PEM as control

^*c*^ The clusters (expression pattern) that proteins are ascribed

^*d*^ Proteins that have no GO annotations

### Hierarchical clustering analyses of temporal protein expression profiles during somatic embryo development

**Fig 3:**
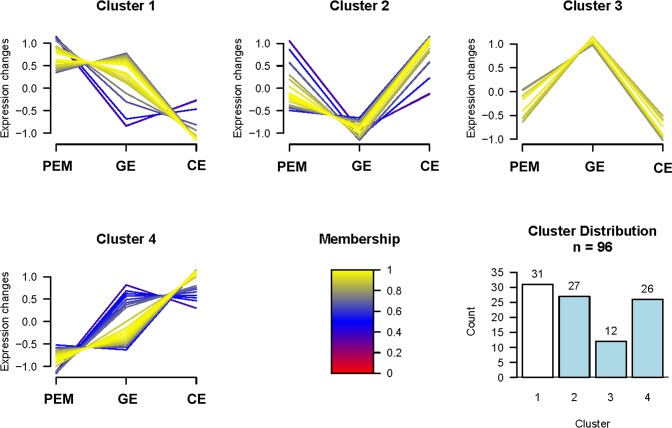
Cluster analysis of proteins differentially expressed during SE. Four clusters were generated to classify proteins during three time points: PEM stage, GE stage and CE stage. The membership values are used to assess how well a given entry fits the consensus profile and allows color coding cluster graph items according to their goodness of fit to the cluster consensus profile.

Regulation patterns typically reflect specific involvement of proteins in corresponding cellular pathways and therefore could serve as a potential clue to the functional roles of these proteins. To better understand the expression patterns of proteins that exhibited significant changes during *L*. *principis-rupprechtii* somatic embryo maturation, proteins that shared the same expression profiles were grouped into four clusters (clusters 1–4), and the numbers of proteins assigned to each cluster were reported (Fig. 3). The membership values were used to assess how well a given entry fit the consensus profile and allowed cluster graph items to be color-coded according to their goodness of fit to the cluster consensus profile. An additional file lists the proteins by cluster number with corresponding log2-transformed ratio metric data and membership values for each protein entry (S2 Dataset).

The largest group, cluster 1, contained 31 proteins whose expression patterns showed a slight decrease from the PEM stage to the GE stage and then a marked drop in expression during the transition between the GE and CE stages. Cluster 2 included 27 proteins exhibiting an intermediate initial expression level that decreased to a minimum during the GE stage and increased subsequently during the CE stage to levels exceeding their initial expression. Cluster 3 was the smallest group with only 12 proteins, but these proteins exhibited the best fit to the cluster consensus profile. The members of this cluster showed marked changes in expression during both developmental stage transitions with low initial expression levels that increased sharply to a maximum during the GE stage and then decreased dramatically during the CE stage. Cluster 4 included 26 proteins that exhibited a gradual rise in expression over the entire course of the *L*. *principis-rupprechtii* somatic embryo development process.

### Functional classification of differentially expressed proteins

**Fig 4:**
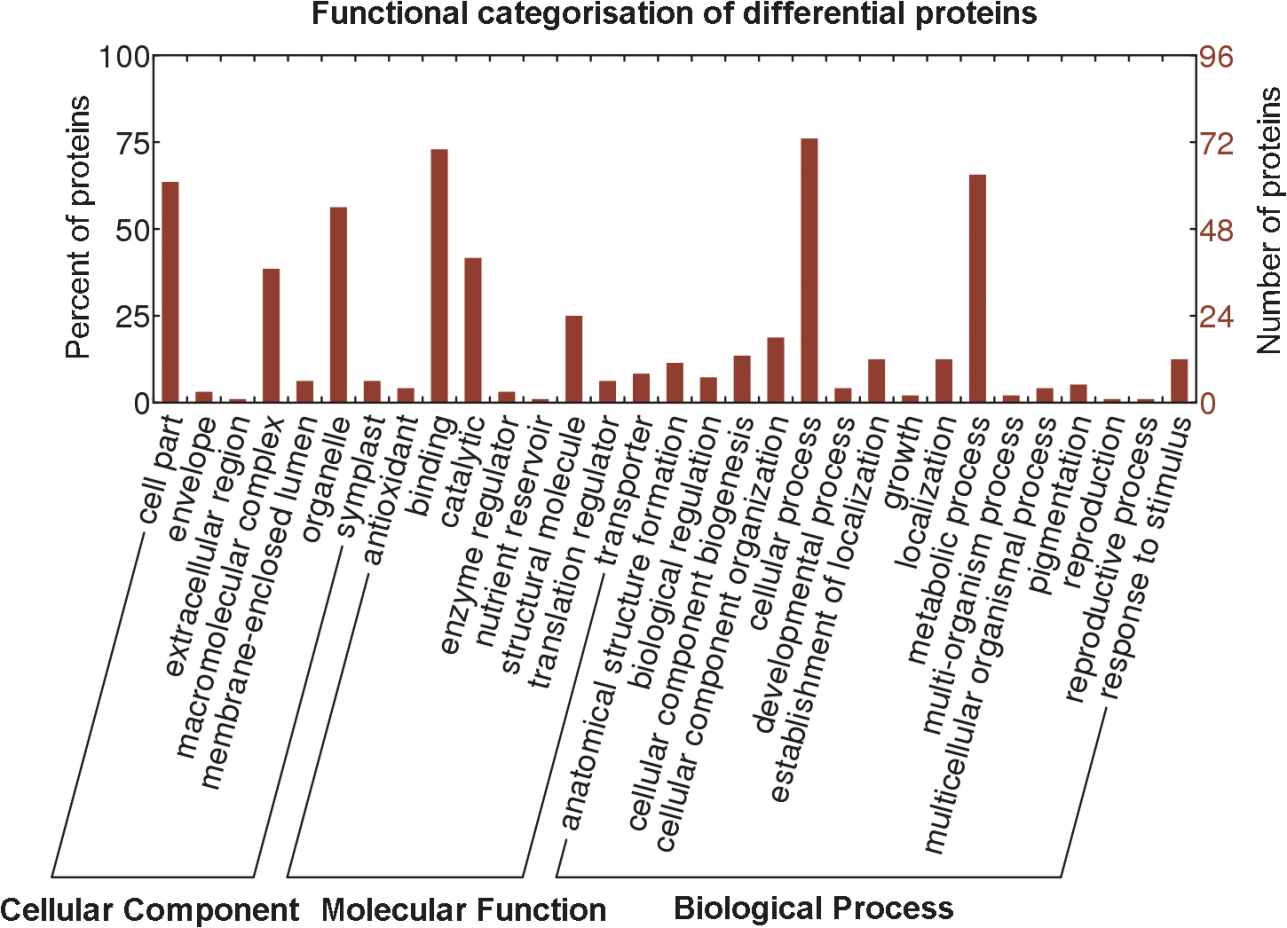
Functional categorization of differentially expressed proteins during SE. Ninety-six differentially expressed proteins during three developmental stages (PEM, GE and CE) were categorized based on “Cellular Component,” “Molecular Function,” and “Biological Process” using WEGO.

Of the 96 proteins with significant levels of differential expression, 90 were assigned GO annotations and were categorized functionally using WEGO, a freely available online application that groups proteins based on their GO annotations. In this study, proteins were categorized at a GO annotation level of greater than or equal to 2 based on the cellular component (CC), molecular function (MF), and biological process (BP) classes with 7, 8, and 16 functional categories, respectively, selected to cover the entire data set. Some proteins were counted more than once if the protein was assigned to more than one category. An overview of the functional categories and the percentage and number of proteins allocated to each function is presented in Fig. 4. Among the enriched categories based on BP, proteins involved in metabolic processes and cellular processes were well represented at 65.6 and 76.0%, respectively, of the differentially expressed proteins. Of the MF category proteins identified in this study, proteins involved in binding and catalytic activity were in the majority, accounting for 72.9 and 41.7%, respectively. In the CC class, GO terms were enriched for the cell and organelle parts with 63.5 and 56.3%, respectively. Proteins involved in developmental cytoskeleton organization and binding accumulated during the early stage of *L*. *principis-rupprechtii* somatic embryo development from the PEM stage to the GE stage. GO terms that were overrepresented in the PEM stage, decreased during the GE stage, and then increased during the CE stage included proteins involved in nucleosome assembly and gene expression. GO terms overexpressed only during the GE stage included proteins involved in transport. Finally, proteins that increased from the PEM stage to the CE stage and accumulated in the latter stage were involved in phosphorylation and oxidation reduction.

### Water content of SE cultures

**Fig 5:**
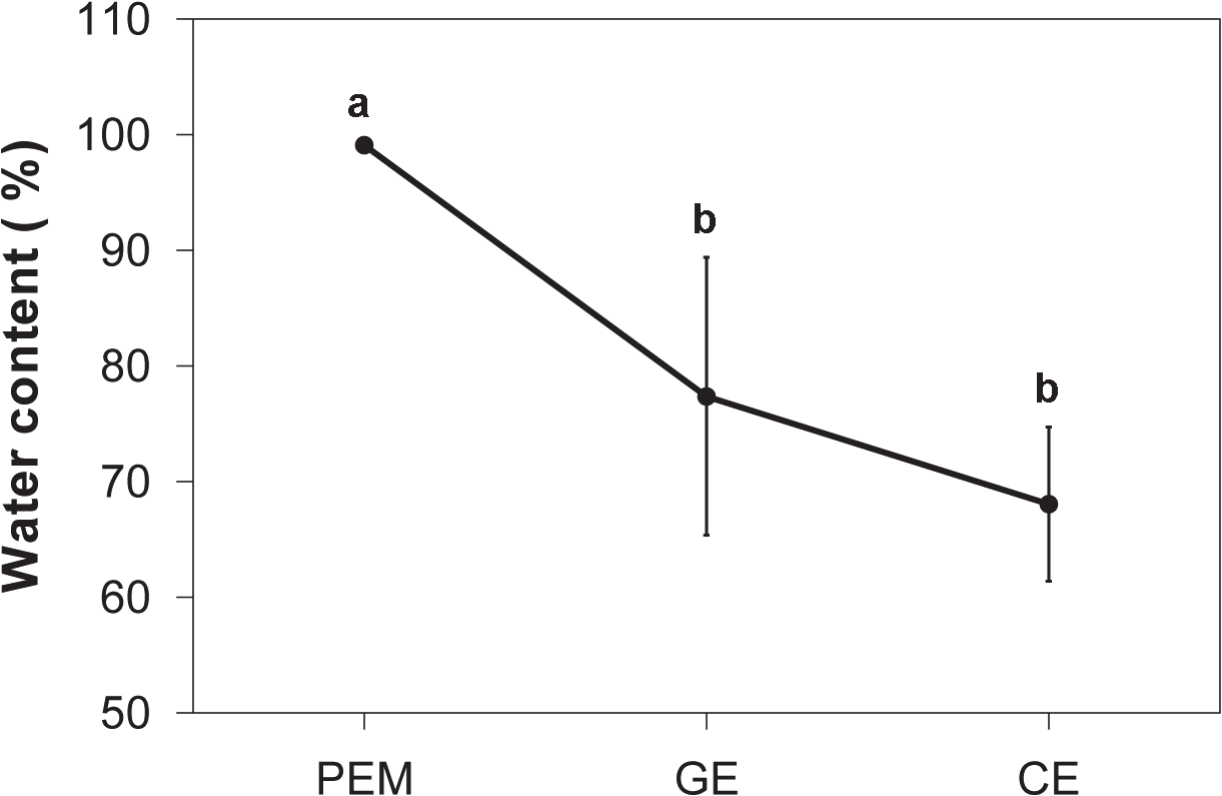
Water content changes during somatic embryo development. Percentage of water content during SE was calculated as (FW-DW)/FW. Bars represent standard errors (n = 5). Significantly different groups are indicated by different lowercase letters (*P* < 0.05).

To characterize the water status during SE development, the water content of three developmental stages were determined (Fig. 5). Overall, the water content decreased significantly during three developmental stages. At early stage, PEMs had quite high water content (99.10±0.06%). When cultures were transformed to maturation medium, their water contents were dropped significantly at GE stage (77.38±12.01%), and with a continued decline at CE stage (68.07±6.67%).

### Enzyme activity assays

**Fig 6:**
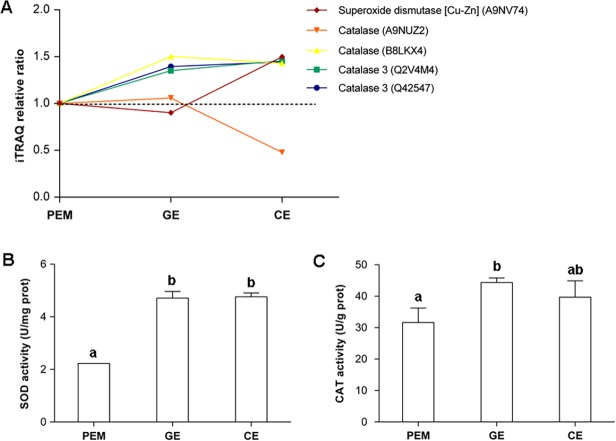
Comparison and validation of SOD and CAT by iTRAQ profiling and enzyme activity assays. iTRAQ analysis showed quantitative changes of SOD and CAT during three developmental stages (A). Activity assays for SOD and CAT were conducted to validate the iTRAQ results (B and C, respectively). Bars represent standard errors (n = 3). Significantly different groups are indicated by different lowercase letters (*P* < 0.05).

Since the iTRAQ data indicated that oxidation-reduction is one of the most responsive pathways during somatic embryo development (Fig. 6A), SOD and CAT assays were performed to further validate our iTRAQ result. As shown in Fig. 6B, total SOD activities increased by 112% from PEM to GE, but no significant changes occurred from GE to CE (Fig. 6B). At GE and CE stages, total CAT activities increased by 40% and 25%, respectively (Fig. 6C), these showing a good correlation with the iTRAQ data.

## Discussion

In this study, we employed an iTRAQ-based shotgun proteomic approach to study somatic embryo development in *L*. *principis-rupprechtii* and identified many differentially expressed proteins. The grouping of the proteins into clusters combined with functional classification analysis gave clear evidence for the involvement of different characteristic temporal patterns of protein expression during *L*. *principis-rupprechtii* somatic embryo development. Expression profile analysis of these proteins revealed key molecular characteristics of the developmental process and identified important clues about the crucial proteins and their co-regulation during specific developmental stages.

**Fig 7:**
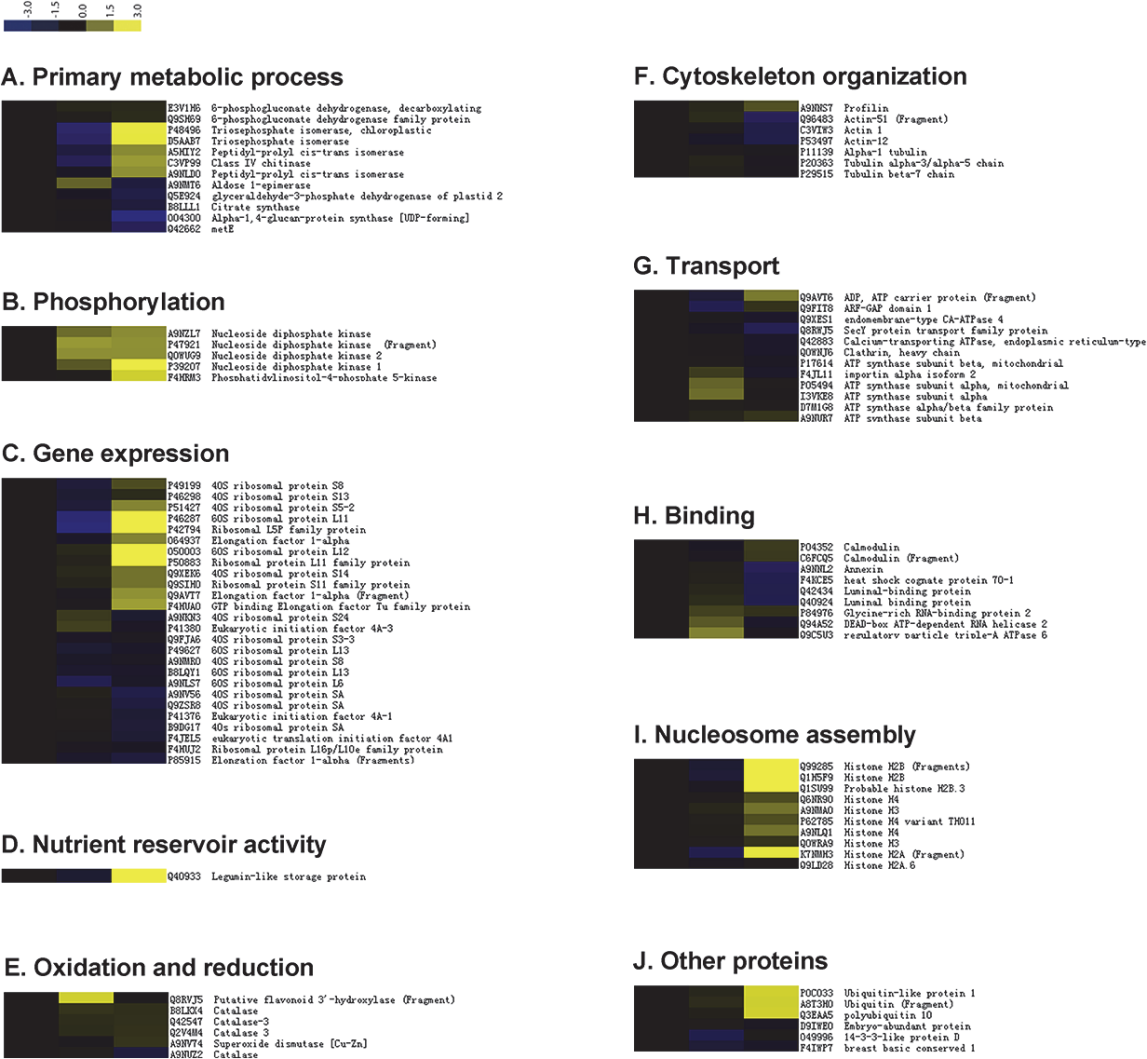
Cluster profiles of protein functional clusters during somatic embryo development. A heat map of the log 2 relative abundance of proteins during SE in relation to the PEM stage was created with the iTRAQ-derived quantitative data. Proteins were grouped according to their known or putative biological functions. For each protein, accession number and protein description are provided. Yellow indicates upregulation, blue denotes downregulation, and black signifies zero difference. The grading represents the ratios of protein expression levels.

Our earlier study had focused on the beginning of SE in *L*. *principis-rupprechtii*, making a proteomic comparison between embryogenic and non-embryogenic tissues [23]. As a continuation, this work was carried out to indicate some important clues not only in the beginning, but also in the whole process of SE. Similarly to previous proteomic study on *Picea glauca* and *Larix × eurolepis* [19,21], some important proteins, such as storage proteins and heat shock proteins (HSPs), were found accumulation and may play crucial roles during this process. Below, the most important proteins related to SE are discussed based on their putative biological functions (Fig. 7).

### Primary metabolic processes

SE is accompanied by active primary metabolic processes (Fig. 7A), especially those related to carbon and nitrogen metabolism. Two glycolytic enzymes, triosephosphate isomerase (TIM; D5AAB7, P48496) and glyceraldehyde 3-phosphate dehydrogenase (GAPDH, Q5E924), and one enzyme involved in the tricarboxylic acid cycle (TCA cycle), citrate synthase (B8LLL1), exhibited changes in expression during somatic embryo development. While TIM expression was downregulated during the GE stage and then upregulated sevenfold, the expression of GAPDH and citrate synthase decreased slightly during maturation. Glycolysis and the TCA cycle are essential for the supply of energy and the formation of metabolic intermediates as reported previously for several species [25,26]. The high level of TIM expression led us to hypothesize that increased energy levels were required for various cellular processes during somatic embryo maturation despite the slight decrease in GAPDH and citrate synthase expression. Similarly, the expression of 6-phosphogluconate dehydrogenase (E3V1H6, Q9SH69), an enzyme participating in the oxidative branch of the pentose phosphate pathway (PPP) that provides NADPH and pentose for multiple metabolic pathways also increased progressively during somatic embryo development. The expression of another galactose metabolism-related enzyme, aldose 1-epimerase (A9NMT6), increased by up to twofold during the GE stage relative to the PEM stage. Alpha-1,4-glucan protein synthase (O04300) is a glycosyltransferase that builds up alpha-1,4-glucan chains that are covalently bound to protein, thus acting as an initiator of glycogen synthesis. The inactivation of alpha-1,4-glucan protein synthase observed in this study was expected due to the increased energy demands of somatic embryo maturation. A pathogenesis-related (PR) protein, chitinase IV (C3VP99), accumulated during the CE stage, similar to results reported previously for Norway spruce [27], indicating that chitinases regulate the differentiation of somatic embryos from PEMs by promoting programmed cell death (PCD).

A protein involved in methionine metabolism and ethylene biosynthesis, 5-methyltetrahydropteroyltriglutamate-homocysteine (MetE, Q42662), was also identified as differentially expressed in this study. This enzyme is likely to be involved in the biosynthesis of l-methionine, which can be transformed into S-adenosylmethionine (SAM), the precursor of ethylene. Unsurprisingly, MetE expression was repressed during somatic embryo maturation due to the negative effect of ethylene on somatic embryo development [28]. Another enzyme, peptidyl-prolyl *cis*–*trans* isomerase (PPIase; A5HIY2 and A9NLD0), which catalyzes *cis*–*trans* isomerization of imide bonds in peptides and proteins [29], was downregulated during the transition between the PEM and GE stages, and then upregulated threefold during the CE stage.

### Phosphorylation

Several enzymes involved in phosphorylation were identified, which consistently fell into cluster 4 and exhibited a tendency for increasing expression during somatic embryo development (Fig. 7B, Table 1). The expression of four nucleoside diphosphate kinases (NDPKs) increased sharply by two- to threefold during the transition from the PEM stage to the GE stage followed by a slight change in expression between the GE and CE stages for three of the NDPKs (Q0WUG9, A9NZL7, P47921) and a greater than sevenfold increase in expression relative to expression during the PEM stage for the remaining NDPK (P39207). NDPKs are essential and ubiquitous enzymes that catalyze the transfer of γ-phosphate from ATP to cognate (d)NDPs to generate (d)NTPs via the phosphorylation of a histidine during the catalytic cycle [30]. Thus, NDPKs maintain the intracellular nucleoside triphosphate pools. Evidence indicates that NDPKs regulate a variety of cellular processes including proliferation, development, and differentiation modulated by signaling pathways, which appears to support the positive role of NDPKs in somatic embryo development. Another differentially expressed phosphorylation-related enzyme identified in this study was phosphatidylinositol-4-phosphate 5-kinase (PIP5K), which participates in the phosphoinositide pathway. In contrast to its expression in coffee plants [31], PIP5K exhibited a significant increase in expression during maturation, reaching a maximum increase of almost sixfold during the CE stage. Previously, PIPK had been reported to be required for actin remodeling [32] and to play a crucial role in the transduction of signals induced by various stimuli [33]. Based on these results, we propose that the progressive increase in PIPK5 expression may be related to responses to stimuli *in vitro* and to the developmental morphogenesis of somatic embryos.

### Gene expression

Physiological and metabolic changes during SE require the synthesis, assembly, and stabilization of newly synthesized proteins as well as the modification and/or removal of peptides [14]. One of the largest functional groups identified in this study consisted of proteins involved in protein biosynthesis and included ribosomal proteins, initiation factors, and elongation factors. Ribosomes, the large protein–RNA machines in all cells, use genetic information to synthesize proteins in the process known as translation [34]. As many as 19 ribosomal proteins were detected (Fig. 7C), some of which exhibited increases in expression as great as 11-fold during the CE stage. Eukaryotic translation is very complex and highly regulated, especially during initiation, which requires many initiation factors to facilitate the scanning of messenger RNAs and the initiation of protein synthesis [35]. Three eukaryotic initiation factors (P41380, P41376, and F4JEL5) and four elongation factors (O64937, Q9AVT7, P85915, and F4HUA0) were expressed differentially during SE. The accumulated evidence indicates that protein metabolism is a key factor in SE, especially during the late stages of embryo development.

### Nutrient reservoir activity

Storage proteins are known to accumulate in plant embryonic tissue during maturation and function as a nutrient reservoir (Fig. 7D) that is required to support the organism until it switches fully to autotrophic growth [19]. In some plant species, the abundance of storage proteins has been used to distinguish zygotic embryos from somatic embryos, which exhibit little or no accumulation of storage proteins [17]. In contrast, legumin-like storage protein, a typical storage protein belonging to the globulin protein family, accumulated to high levels during the late stages of SE, probably in response to partial dehydration during somatic embryo development, just as the water content assay shown (Fig. 5).

### Oxidation reduction

Overproduction or accumulation of reactive oxygen species (ROS) can disturb the redox system in cells and cause damage to proteins, lipids, and nucleic acids, thereby compromising cell viability [36,37]. Plant cells can defend against ROS by means of antioxidant enzymes that regulate the levels of ROS to reduce ROS damage [38]. Superoxide dismutase (SOD) acts as a first line of defense against ROS by dismutating superoxide to H_2_O_2_, thereby decreasing the risk of hydroxyl radical formation from superoxide [39]. Catalase (CAT) plays a principal role in ROS scavenging in plants. In agreement with results from other species, differential expression of SOD and CAT during somatic embryo induction was observed in both the iTRAQ analysis and enzyme activity assays, with a tendency for a progressive increase in expression during maturation (Table 1; Fig. 6) due in part to increased ROS after exposure to ABA or high osmotic stress.

Flavonoid 3'-hydroxylase (Q8RVJ5) is involved in the flavonoid biosynthesis pathway, and its high expression at the globular stage may indicate the accumulation of flavonoids during the GE stage. Flavonoids have been shown to act as antioxidants [40] and as inhibitors of polar auxin transport [41] in higher plants. The transition from PEMs to somatic embryos is known to depend on the degradation of suspensor cells resulting from PCD induced by the withdrawal of auxin [42]. Therefore, we concluded that the accumulation of flavonoid 3'-hydroxylase during the globular stage may have been due to the withdrawal of auxin during the transition between the early developmental stages and to the scavenging of ROS when PEMs were exposed to a new environment.

### Cytoskeleton organization

Embryogenesis is accompanied by distinct morphogenesis based on dynamic cytoskeleton reorganization. The plant cytoskeleton consists of microtubules and microfilaments, two dynamic interconnected arrays assembled from tubulins and actins. We identified three tubulins (P29515, P20363, and P11139) and three actins (Q96483, C3VIW3, and P53497) that exhibited dynamic expression profiles during somatic embryo development (Fig. 7F). Tubulins are known to be involved in cell division and elongation and to play an important role in the separation of the organelles and daughter chromosomes during mitosis [43]. Accumulated evidence indicates that depolymerization of the actin cytoskeleton acts as a downstream regulatory factor in plant stress adaptation networks and participates in the initiation and regulation of PCD [44,45]. Therefore, we expected to detect dynamic expression of tubulins and actins during somatic embryo development as shown previously in other species [13,43]. In this study, we found that profilin accumulated during SE. Profilins are multifunctional proteins involved in signal transduction and the linkage between the plasma membrane and the actin cytoskeleton, as well as in organelle location within the actin cytoskeleton [46]. Profilin accumulation is thought to play an active role in cytoskeleton organization as part of somatic embryo development.

### Transport proteins

Transport proteins accounted for large part of the differentially expressed proteins identified in this study and possessed functions related to transmembrane and intracellular trafficking and nutrient transport. The expression of several ATP synthase subunits (P05494, P17614, I3VKE8, A9NUR7, and D7M1G8) was upregulated during the GE or CE stages. ATP synthases are key enzymes present in mitochondria, chloroplasts, and photosynthetic bacteria that participate in oxidative phosphorylation and photophosphorylation to provide cells with ATP. Moreover, the ATP synthase β-subunit was reported recently to play a role in PCD and to be a novel target for extracellular ATP in its function as a key negative regulator of PCD [47]. Based on these prior findings, we expected high expression of ATP synthase during certain stages of somatic embryo maturation. ADP-ribosylation factor GTPase-activating proteins (ARF-GAPs; Q9FIT8) were upregulated during the CE stage. ARF-GAPs are best known as regulators of membrane traffic that control the assembly and disassembly of vesicle coat proteins and for their involvement in remodeling the actin cytoskeleton as cells change shape or move [48]. Importin (F4JL11) and ADP/ATP carrier protein (Q9AVT6) were also expressed at high levels during the GE or CE stages.

Several transport proteins including Ca^2+^-transporting ATPases (Q9XES1 and Q42883), clathrin (Q0WNJ6), and SecY transport protein (Q8RWJ5) exhibited decreased expression during SE in *L*. *principis-rupprechtii*. Ca^2+^-transporting ATPases are localized in endomembranes or the plasma membrane and play a key role in removing Ca^2+^ from the cytoplasm to terminate signaling events, a process that is critical for Ca^2+^ homeostasis in all eukaryotic cells [49]. The Sec protein translocation channel serves as the platform needed to bring together the many different components required for the constitutive and obligatory process of protein transport to create a ubiquitous protein-conducting pathway by which thousands of newly synthesized polypeptides make their way through the lipid bilayer [50]. Clathrin has been shown to play a fundamental role in endocytosis and auxin-mediated development *in planta* [51]. Further investigation into their roles during SE is needed.

### Binding proteins

Binding proteins comprised a large group of the differentially expressed proteins identified in this study (Fig. 7H), indicating their crucial roles during SE in *L*. *principis-rupprechtii*. Exogenous Ca^2+^ and the maintenance of cellular Ca^2+^ gradients are known to be required for proper embryo development *in vitro* [52]. In this study, two calmodulin (CAM; P04352 and C6FCQ5) calcium-sensing proteins involved in ion transport, metabolism, transcriptional regulation, protein phosphorylation, and other critical functions [53] exhibited a slight decrease in expression during the GE stage followed by increased expression during the CE stage. The accumulation of CAM during the CE stage may be due to increased demand for Ca^2+^ involved in active Ca^2+^-mediated signaling to promote embryo development.

Annexins are multifunctional proteins expressed throughout the life cycle that appear capable of linking Ca^2+^, redox reactions, and lipid signaling to coordinate development in response to the biotic and abiotic environments [54]. They interact with various cell-membrane components associated with intracellular signaling and cell structural organization and growth regulation [55]. In this study, annexin (A9NNL2) expression increased slightly during the GE stage and decreased during the CE stage.

Stress response proteins are frequently reported to be present in dividing cells or tissues, among which heat-shock proteins (HSPs) were found to be the most abundant during SE [18]. Most HSPs are known as molecular chaperones that assist in refolding proteins under stress conditions or stabilize the unfolded state of newly synthesized proteins to prevent them from misfolding or aggregating. Several HSP family proteins including heat-shock cognate protein 70–1 (HCP 70–1; F4KCE5) and luminal-binding proteins (BiPs; Q42434 and Q40924) were upregulated markedly between the PEM and GE stages, and downregulated during the CE stage. HSPs were reported previously in many studies to be present throughout embryogenesis [21,56]. Likewise, BiPs have been reported to play a role in binding nascent polypeptides to induce correct folding and seed development [57]. We hypothesize that high expression of HSPs during the globular stage is part of a general stress adaptation process that implies a fine regulation of auxin and stress signaling when PEMs are transferred to ABA-containing hypertonic medium.

Glycine-rich RNA-binding proteins (GRPs; P84976) are a class of RNA-binding proteins involved in general molecular responses to environmental stress that are mediated by posttranscriptional regulatory mechanisms. Interactions between GRPs and DEAD-box ATP-dependent RNA helicase have been reported [58]. Consistent with these prior results, both of these proteins accumulated during the globular stage in association with the transition to different medium in this study.

### Nucleosome assembly

Our results indicated an active role for histones during somatic embryo development. Ten forms of histones, including H2A, H2A.6, H2B, H2B.6, H3, and H4 and a histone H4 variant THO11 involved in nucleosome assembly (Fig. 7I), were identified in this study and grouped into clusters 2 and 4 (Table 1). They exhibited expression patterns of downregulation or slight upregulation from the PEM to GE stages and then displayed a sharp increase to greater than tenfold higher levels during the CE stage. Previously, histones were shown to be expressed differentially during SE in *C*. *persicum* [15]. Histones are prone to reversible posttranslational modifications such as methylation, phosphorylation, glycosylation, acetylation, and ubiquitination [59]. A quantitative study on posttranslational modifications of histones would reveal more information on the regulation of SE.

### Other proteins

The last group consisted of those proteins that did not have GO annotations based on their biological functions in UniprotKB (Fig. 7J), but their roles in the process of SE in *L*. *principis-rupprechtii* should not be underestimated. Ubiquitin is a common denominator in the targeting of substrates to protein degradation pathways [60] and ubiquitin expression increased during SE, especially during the CE stage. Protein breakdown may play a key role in somatic embryo development by degrading proteins of specific pathways and subsequently supplying amino acids for the biosynthesis of new proteins. 14–3–3 proteins regulate a wide range of target proteins in all eukaryotes through phosphorylation in response to environmental, metabolic, and nutritional stresses as well as in defense responses to wounding and pathogen attack [61]. 14–3–3 proteins have been shown to play negative roles in the regulation of ATP synthase [62], which may account for the low expression of 14–3–3 proteins during the GE stage consistent with the high expression of ATP synthase during this stage. In addition, differential expression of embryo-abundant protein was also detected in this study with a slow decline during SE.

## Conclusions

This work is the first application of iTRAQ technique to detect proteome changes during somatic embryo development in *L*. *principis-rupprechtii*. This study determined quantitative changes in protein expression profiles in developing somatic embryos and showed their functional relationships in the process of SE. Our results indicate increasing metabolic activity during SE as well as the activation of phosphorylation-related enzymes such as NDPK and PIP5K. The detection of differential expression of legumin-like storage protein builds on existing knowledge and reinforces the importance of its role in embryogenesis. Moreover, the high expression of binding proteins involved in response to stress, such as annexin, HCP, and BiP, indicates a general stress adaptation process that implies a fine regulation of auxin and stress signaling when PEMs are transferred to ABA-containing hypertonic medium. These proteins may be potential candidates for further investigation in terms of their biology and functional relevance to SE, such as the posttranslational modification of histones. This work provides novel insights into the process of larch embryo development and a framework for further study of the biological process and opportunities for practical application of this knowledge.

## Supporting Information

S1 Dataset Details for identified proteins and peptides. (XLS)

S2 Dataset Details for differentially expressed proteins that grouped into four clusters. (XLS)
